# The effectiveness of HPV16 and HPV18 genotyping and cytology with different thresholds for the triage of human papillomavirus-based screening on self-collected samples

**DOI:** 10.1371/journal.pone.0234518

**Published:** 2020-06-11

**Authors:** Fangbin Song, Hui Du, Chun Wang, Xia Huang, Ruifang Wu, CHIMUST team

**Affiliations:** 1 Department of Obstetrics and Gynecology, Peking University Shenzhen Hospital, Shenzhen, Guangdong, PR China; 2 Shenzhen Key Laboratory on Technology for Early Diagnosis of Major Gynecological Diseases, Shenzhen, Guangdong, PR China; Penn State University School of Medicine, UNITED STATES

## Abstract

**Background/Objective:**

Human papillomavirus (HPV) genotyping and cytology have been recommended for colposcopy triage, but it is unclear which combinations of high-risk HPV (hrHPV) types and cytology with various thresholds provide clinically useful information for the triage after primary HPV screening on self-collected samples.

**Method:**

Chinese Multi-site Screening Trial (CHIMUST) database focused on self-collected samples was reviewed using the results of Cobas4800 HPV assay. Absolute risks of each genotype for cervical intraepithelial neoplasia 2 or worse/ 3 or worse (CIN2+/CIN3+) were calculated. Triage of atypical squamous cells of undetermined significance (ASCUS) or worse cytology was used as the comparator, and diagnostic accuracy for paired comparisons between algorithms was obtained using McNemar’s test.

**Results:**

A total of 10, 498 women were included, the overall prevalence of hrHPV, HPV16, HPV18, and Other hrHPV genotypes were 13.7%, 2.4%, 0.8%, and 10.5%, respectively. HPV16-positive women had the highest absolute risk among various genotypes for CIN2+/CIN3+ whether in normal or abnormal cytology (ASCUS or worse) and among all age groups. When compared with the comparator, combining HPV16 positivity and/or high-grade squamous intraepithelial lesion (HSIL) or worse yielded higher specificity (97.7% vs. 97.0%, p<0.0001), similar sensitivity (90.7% vs. 96.3%, p = 0.256) for detection of CIN3+, and a decrease in colposcopy referral rate from 3.5% to 2.7%, similar results were found for CIN2+. Positivity for HPV16 and/or (ASCUS or worse), and positivity for (HPV16 and/or HPV18) and/or (ASCUS or worse) achieved favorable sensitivity compared with the comparator (80.6% and 81.3% vs. 70.1% respectively for CIN2+, p<0.0001; both 96.3% vs. 96.3% for CIN3+, p = 1.000), these algorithms would reduce the colposcopy referral rate to 5.0% and 5.6% respectively, compared with 13.7% of that for HPV alone.

**Conclusions:**

Triage of HPV-positive women on self-collected samples by combining HPV16 or HPV16/18 genotyping with different thresholds of cytology could provide tradeoffs in sensitivity for detecting cervical lesions and colposcopy referral rates, and tailor management in various circumstances of clinical practice.

## Introduction

Cervical cancer has caused estimated 311,365 deaths worldwide in 2018, with 90% occurring in developing countries[1]. Although three Human papillomavirus (HPV) vaccines have been approved successively in mainland China since 2016, none of them has been incorporated into the National Immunization Program yet. Accordingly, despite the effectiveness of vaccines, nearer-term impact will require delivery of cervical screening to older cohorts who will not benefit from HPV vaccination[2].

HPV test has been proved as a cost-effective primary screening method worldwide [3, 4], which could increase the coverage of screening via self-collected samples [5]. In low-resource areas without the infrastructure to support a cytology-based system, HPV testing can be done on self-collected samples, and this may offer opportunities to reach those who are reluctant to undergo gynecological examinations. Since self-collected sampling greatly reduces the physician workload for cervical cancer screening it can reduce cost and at the same time achieve better coverage in low-resource areas.

Most women find HPV self-sampling to be more convenient, less embarrassing, and less painful than clinician-sampling, but are concerned about test accuracy[6]. When used with high-risk HPV (hrHPV) assays that amplify the sample, such as polymerase chain reaction (PCR) assays, HPV testing on a self-collected sample is as sensitive as a clinician collected endocervical specimen for the detection of high-grade lesions [7, 8]. However, the management of HPV positive patients remains challenging due to the relatively low specificity of HPV testing in general[9].

Cytology and genotyping are the most common methods used for triage of HPV positive patients [9]. Cytological triage of a positive HPV test has been primarily proposed as an appropriate method of reducing colposcopy referrals. However, according to the Addressing the Need for Advanced HPV Diagnostics HPV Study (ATHENA trial), strategies that depended on cytology for triage of HPV-positive women decreased the sensitivity of HPV test[10]. Due to the differing risks associated with different genotypes [11, 12], partial types such as HPV16 and HPV18 are widely reported and used to guide clinical management[13]. Incorporating screening with HPV and triage of HPV-positive women by a combination of genotyping for HPV16/18 and cytology provided a good balance between maximizing sensitivity and specificity by limiting the number of colposcopies[10]. However, it is unclear what combinations of genotyping for HPV16, or HPV16/18 with various thresholds of cytological grades provide clinically useful information for the triage after primary HPV screening.

In the present study, we reviewed the Chinese Multi-site Screening Trial (CHIMUST) database, and focused on self-collected samples, to explore the performance of HPV16 alone or HPV16/18 combined with different cytological cut-offs as methods of risk-stratification to triage patients after primary HPV screening.

## Materials and methods

### Subjects and study design

We reviewed the data from CHIMUST, a population-based study that was conducted in Beijing and 5 Provinces in China from Aug 2016 to Jan 2018 (The Trial Registration Number: ChiCTR-EOC-16008456). A total of 10,885 non-pregnant women, aged 29 to 60 years, with an intact uterus, without history of pelvic radiation, and without cervical cancer screening within 3 years were recruited. Briefly, CHIMUST is a primary HPV testing cervical cancer screening trial, all self-collected and clinician-collected samples were tested with the PCR-based hrHPV assays: Cobas4800 and SeqHPV (BGI, Shenzhen, China). Patients with HPV-positive results from any HPV assay on either self-collected or clinician-collected samples were randomly referred to colposcopy and biopsy. Cytology was used only for analysis and not for colposcopy referred requirements (S1 Fig). This study is a nested substudy of the CHUMUST trial, focusing on Cobas HPV assay on self-collected samples. Women with complete data on Cobas HPV on self-collected samples, cytology and histological results were analyzed. The CHIMUST protocol was approved by the Ethics Committee from the Peking University Shenzhen Hospital (PUSH), Shenzhen, China, as well as the current study (No. 2016001). All participants signed an informed consent before enrollment. Information that could identify individual participants was fully anonymized during or after data collection.

### Self-sample collection and HPV DNA detection

All women who participated in the screening provided a self-collected vaginal sample. The participants were requested to place the sampling brush into the top of the vagina and then rotate it 3 times. After sampling, the brush with specimen was rubbed on a solid specimen processing (SSP) card, followed by placing it into a small bottle containing 6mL of PreservCyt^®^ solution (Hologic, Marlborough, Mass. USA). Self-collected samples were prepared for the Cobas4800 assay (Roche) per the manufacturer’s instructions. Results from Cobas4800 testing are either HPV16 positive, HPV18 positive, other 12 types of hrHPV (Other hrHPV) positive, or HPV negative. Hierarchical typing results ranked as HPV16, HPV18, Other hrHPV, and negative.

### Clinician-sample collection and liquid based cytology

After self-sampling, cervical exfoliated cell samples were obtained at the squamocolumnar junction of the cervix by prior-trained clinicians with a sampling brush which was subsequently placed into a 20mL PreservCyt^®^ solution for testing on Cobas4800, SeqHPV, and cytology test (ThinPrep, Hologic). Cytology slides were interpreted by senior cytopathologists in PUSH according to the Bethesda classification system [14].

### Colposcopy, biopsy and histological diagnoses

Patients with HPV positive results from any HPV assay in CHIMUST (Cobas4800, SeqHPV on either self-collected or clinician-collected samples) were called back for colposcopy and biopsy. Colposcopy-directed biopsies were performed according to the Preventive Oncology International (POI) protocol [5]. Histological slides were analyzed by PUSH pathologists blind of any other testing results and the results were reported in 5 categories as negative, cervical intraepithelial neoplasia (CIN) 1, CIN 2, CIN 3, and cancers. In patients who had more than one tissue sample, the highest diagnosis was recorded. Women with atypical squamous cells of undetermined significance (ASCUS) or normal cytology and negative hrHPV results were considered to have a minimal risk of CIN and were classified as within normal limits[5].

### Statistical analysis

Histologically confirmed CIN3 or worse (CIN3+) and CIN2 or worse (CIN2+) were used as the study endpoints. The absolute risks (ARs) of high-grade cervical disease were determined for different HPV types, and the respective 95% confidence intervals (CIs) for ARs were estimated by bootstrapping (1,000 times) [15]. Combinations of genotyping for HPV16, or HPV16/18 with different thresholds of cytological grades (≥ASCUS, low-grade squamous intraepithelial lesion or worse [≥LSIL], or high-grade squamous intraepithelial lesion or worse [≥HSIL]) were evaluated to identify potentially better algorithms for triage to immediate colposcopy in reference to the comparator, being cytology with a threshold of ASCUS [16]. Sensitivity, specificity, colposcopies referred rates, and number of colposcopies and cytology tests required to detect one case of CIN2+/CIN3+ were calculated. Exact p-values of diagnostic accuracy for paired comparisons between algorithms were obtained using McNemar’s test. Analyses were carried out using SPSS software 24.0 (IBM Company, Chicago, IL). All analyses were two-sided, and *P* values of <0.05 were considered statistically significant.

## Results

### Characteristics of study population

**Fig 1:**
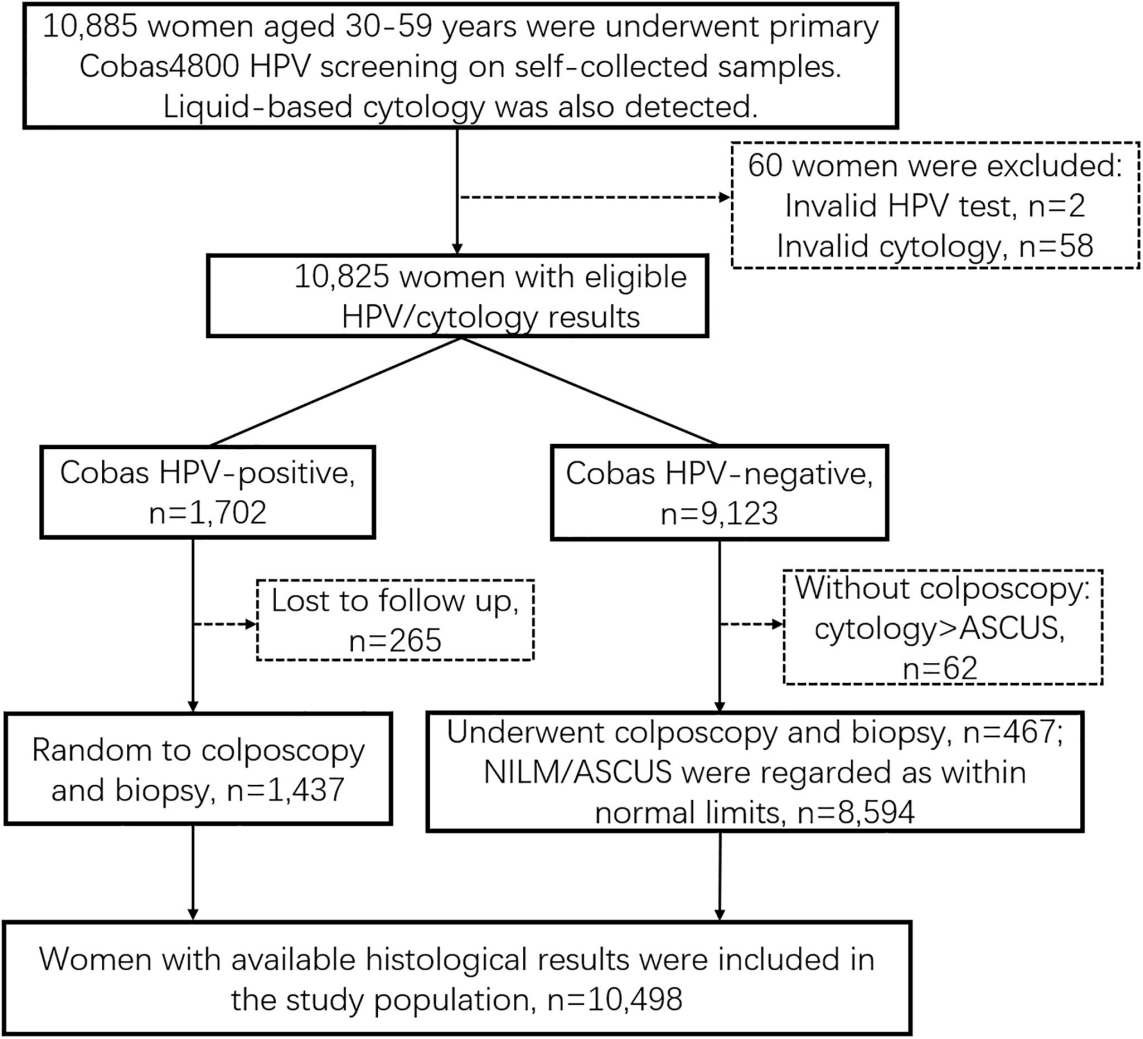
Selection of the study population. NILM, negative for intraepithelial lesion or malignancy; ASCUS, atypical squamous cells of undetermined significance.

Out of 10,885 women offered self-sampling for hrHPV testing in CHUMUST, 58 women were excluded because invalid cytology, and 2 because invalid HPV, 265 because of not having histological results after HPV-positive results, 62 because HPV negative on all assays and cytology≥ LSIL therefore without histopathology, leaving 10,498 cases available for analysis (Fig 1). The rate of compliance after colposcopy referral was 84.4% (1,437/1,702) in HPV-positive women. In the study population of total 10,498 women, the mean age was 44 years (range: 29–60 years). In addition, the majority had normal cytology (9782, 93.2%), 465 (4.4%) had ASCUS, 133 (1.3%) had LSIL or atypical glandular cell (AGC), 39 (0.4%) had atypical squamous cells cannot excluding high-grade lesions (ASC-H), and 79 (0.8%) had HSIL or worse cytology. Moreover, 5 women had a histological result of cervical cancer, 49 were diagnosed with CIN3 or adenocarcinoma in situ (AIS), 90 with CIN2, 159 with CIN1, and 10,212 with no CIN.

### Prevalence of hrHPV on self-samples by age and cytology

Among the 10,498 women, the overall prevalence of hrHPV, HPV16, HPV18, and Other hrHPV genotypes were 13.7%, 2.4%, 0.8%, and 10.5%, respectively (Table 1). The overall prevalence of hrHPV positivity increased with age (P_trend_<0.0001). Among women aged 50 to 60 years old, the prevalence of hrHPV positivity was highest of 15.4%, and the prevalence of HPV16 and/or 18 was highest of 3.7% among various groups (Table 1). Hierarchical typing results were significantly associated with cytological grades (P_trend_<0.0001).

**Table 1:** Prevalence of hrHPV and genotypes on self-samples by age and cytology.

Characteristics	Total	hrHPV results (%)				Chi-square (p-value)
		hrHPV+	HPV16+	HPV18+	Other hrHPV+	
Age: Mean ± SD (Y)	44 ±7.5					1.2 (p = 0.267)
Overall	10498	13.7	2.4	0.8	10.5	
29–39	3310	11.2	2.3	0.6	8.3	
40–49	4396	14.5	2.5	0.6	11.4	
50–60	2792	15.4	2.5	1.2	11.7	
Cytology						48.2 (p<0.0001)
NILM	9782	10.9	1.6	0.7	8.7	
ASCUS	465	33.5	6.5	1.7	25.4	
LSIL/ASC-H/AGC	172	82.6	18.0	2.9	61.6	
HSIL or worse	79	91.1	49.4	3.8	38.0	

NILM, no intraepithelial lesion and malignant cells; ASCUS, atypical squamous cells of undetermined significance; LSIL, low-grade squamous intraepithelial lesion; HSIL, high-grade squamous intraepithelial lesion; AGC, atypical glandular cell; ASC-H, atypical squamous cells, cannot excluding HSIL; SD, standard deviation; hrHPV, high-risk human papillomavirus

### Risk stratification of HPV genotypes for CIN2+/CIN3+

The estimated ARs were dependent on hrHPV types, and HPV16 positivity achieved the highest ARs among various types for CIN2+/CIN3+ whether in normal or abnormal cytology (≥ASCUS) and in all age groups. The ARs for CIN2+ in women with hrHPV positivity/normal cytology, ranged from 1.6% (95% CI = 1.6–6.3%) for women positive of HPV18 to 9.7% (95%CI = 5.2–14.8%) for women positive of HPV16 (Table 2). The ARs for CIN3+ ranged from 1.3% (95%CI = 1.3–5.0%) in women positive of HPV18 to 14.1% (95%CI = 10.2–18.8%) in women positive of HPV16. ARs for various HPV genotypes stratified by age and cytology were shown in Table 2.

**Table 2:** Impact of hrHPV types on estimated absolute risk of CIN2+/CIN3+ (%).

Factors	Overall	Age			Cytology	
		29–39	40–49	50–60	NILM	≥vASCUS
CIN2+						
hrHPV+	9.4 (7.9–10.9)	9.2(6.5–12.4)	10.2 (8.0–12.5)	8.4(5.8–11.2)	3.2(2.2–4.2)	27.3(22.7–31.6)
HPV16+and/or 18+	21.2(17.0–25.4)	23.7(15.5–32.0)	23.7 (16.3–31.1)	15.5(8.7–22.3)	7.3(4.1–11.0)	47.4(38.8–56.9)
HPV16+	26.3(21.2–31.8)	28.9(19.7–39.5)	26.6 (19.3–34.9)	22.9(14.3–32.9)	9.7(5.2–14.8)	52.0(42.0–62.0)
HPV18+	5.0 (1.3–10.0)	4.8 (4.8–19.0)	11.5 (3.8–23.1)	NA	1.6 (1.6–6.3)	18.8 (6.3–37.5)
Other hrHPV+	5.8 (4.5–7.2)	4.0 (1.8–6.6)	6.6 (4.6–8.9)	6.1(3.4–8.9)	2.1 (1.2–3.2)	18.1(13.4–22.4)
CIN3+						
hrHPV+	3.6 (2.6–4.7)	3.2(1.4–5.4)	4.2 (2.8–6.0)	3.0 (1.6–4.7)	NA	14.1(10.8–17.8)
HPV16+and/or18+	11.0(7.8–14.6)	9.3(4.1–15.5)	15.6(9.6–22.2)	6.8(1.9–12.6)	NA	31.9 (23.3–40.5)
HPV16+	14.1(10.2–18.8)	11.8(5.3–19.7)	18.3(11.0–25.7)	10.0 (2.9–17.1)	NA	36.0 (26.0–46.0)
HPV18+	1.3 (1.3–5.0)	NA	3.8 (3.8–13.1)	NA	NA	6.3 (6.3–24.7)
Other hrHPV+	1.4(0.7–2.1)	1.1(0.4–2.6)	1.2(0.4–2.2)	1.8(0.6–3.4)	NA	5.9 (3.1–9.1)

Absolute risk (%) is the number of subjects with disease /number of subjects with positive test results; NILM, no intraepithelial lesion and malignant cells; ASCUS, atypical squamous cells of undetermined significance; CIN2+/CIN3+, cervical intraepithelial neoplasia 2 or worse/ 3 or worse; NA, not applicable; hrHPV, high-risk human papillomavirus; NA, not available

### Prevalence of histological grades by triage tests

Table 3 presented the distribution of histological outcomes according to cytology grades and genotypes. Histological results were available for 1,437 of the hrHPV-positive women. The majority of these women had normal cytology (74.3%) and normal histological results (79.6%). Moreover, prevalence of cytology abnormality (≥ASCUS, ≥LSIL, and ≥HSIL), genotyping for HPV16, and genotyping for HPV16/18 were increased with the aggravation of the histological grades (All P_trend_ <0.0001).

**Table 3:** Distribution of histological results by triage test results among HPV-positive women (n = 1,437).

Triage Results	Histology, n (%)				Total
	Normal	CIN 1	CIN 2	CIN 3+	
**Cytology**					
NILM	925 (80.9)	108(68.4)	34 (41.0)	0 (0.0)	1,067(74.3)
≥ASCUS	219 (19.1)	50 (31.6)	49 (59.0)	52 (100.0)	370 (25.7)
≥LSIL	106 (9.3)	23 (14.6)	37 (44.6)	48 (92.3)	214(14.9)
≥HSIL	12 (1.0)	1(0.6)	20 (24.1)	39 (75.0)	72(5.0)
**HPV genotyping**					
HPV16 positive	163 (14.2)	25 (15.8)	31 (37.3)	36(69.2)	255(17.7)
HPV16 negative	981 (85.8)	133 (84.2)	52 (62.7)	16 (30.8)	1,182(82.3)
HPV16/18 positive	232 (20.3)	32 (20.3)	34 (41.0)	37 (71.2)	335 (23.3)
HPV16/18 negative	912 (79.7)	126(79.7)	49 (59.0)	15 (28.8)	1,102(76.7)
**Total**	1,144	158	83	52	1,437

NILM, no intraepithelial lesion and malignant cells; ASCUS, atypical squamous cells of undetermined significance; LSIL, low-grade squamous intraepithelial lesion; HSIL, high-grade squamous intraepithelial lesion; CIN, cervical intraepithelial neoplasia; CIN3+, cervical intraepithelial neoplasia 3 or worse; HPV, human papillomavirus

### Different triage strategies for the detection of CIN3+ (Table 4) and CIN2+ (Table 5)

We investigated various combinations of genotyping for HPV16, or HPV16/18 with different thresholds of cytology for the triage of HPV-positive women. The characteristics of all 18 strategies (HPV alone and 17 triage strategies) for detecting CIN3+ lesions are detailed in Table 4 and that for detecting CIN2+ lesions are given in Table 5. Triage with cytology testing with an ASCUS threshold (Strategy 2), was used as the comparator, showed a sensitivity of 70.1% (95% CI = 62.0–77.5%), a specificity of 97.4% (95% CI = 97.1–97.7%) for CIN2+. Moreover, the referral rate was 3.5%, and 3.7 colposcopies were needed to find 1 CIN2+.

**Table 4:** Performance of different triage algorithms of HPV positive women for CIN3+ detection.

Triage strategies after primary hrHPV screening	SEN, %	SPE, %	PPV, %	NPV, %	Colposcopy referral rate, %	Cytology test rate %	Colposcopies to detect 1 CIN3+
1. None (all HPV positivity to colposcopy)	96.3	86.7	3.6	100.0	13.7	0.0	27.63
2. ≥ASCUS (comparator)	96.3	97.0	14.1	100.0	3.5	13.7	7.1
3. ≥LSIL	92.6	**98.1**	19.9	100.0	2.4	13.7	5.0
4. ≥HSIL	**75.9**	**99.6**	51.9	99.9	0.8	13.7	1.9
5. HPV16+	**66.7**	**97.9**	14.1	99.8	2.4	0.0	7.1
6. HPV16+ and/or 18+	**68.5**	97.1	11.0	99.8	3.2	0.0	9.1
7. HPV16+ and/or ≥ASCUS	96.3	**95.5**	9.9	100.0	5.0	11.3	10.1
8. HPV16+ and ≥ASCUS	**66.7**	**99.4**	36.0	99.8	1.0	11.3	2.8
9. HPV16+ and/or ≥LSIL	94.4	96.7	12.8	100.0	3.8	11.3	7.8
10. HPV16+ and ≥LSIL	**61.1**	**99.6**	47.1	99.8	0.7	11.3	2.1
11. HPV16+ and/or ≥HSIL	90.7	**97.7**	17.0	100.0	2.7	11.3	5.9
12. HPV16+ and ≥HSIL	**48.1**	**99.9**	66.7	99.7	0.4	11.3	1.5
13.(HPV16+ and/or 18+) and/or ≥ASCUS	96.3	**94.9**	8.8	100.0	5.6	10.5	11.3
14.(HPV16+ and/or 18+) and ≥ASCUS	**68.5**	**99.2**	31.9	99.8	1.1	10.5	3.1
15.(HPV16+ and/or 18+) and/or ≥LSIL	94.4	**96.0**	10.8	100.0	4.5	10.5	9.2
16.(HPV16+ and/or 18+) and ≥LSIL	**63.0**	**99.6**	43.6	99.8	0.7	10.5	2.3
17.(HPV16+ and/or 18+) and/ or ≥HSIL	90.7	97.0	13.4	100.0	3.5	10.5	7.5
18.(HPV16+ and/or 18+) and ≥HSIL	**50.0**	**99.9**	64.3	99.7	0.4	10.5	1.6

Bold value indicates that the value is significantly different from that of the comparator (p<0.01). SEN, sensitivity; SPE, specificity; ASCUS, atypical squamous cells of undetermined significance; LSIL, low-grade squamous intraepithelial lesion; HSIL, high-grade squamous intraepithelial lesion; CIN3+, cervical intraepithelial neoplasia 3 or worse; HPV, human papillomavirus; PPV, positive predictive value; NPV, negative predictive value.

**Table 5:** Performance of different triage algorithms of HPV positive women for CIN2+ detection.

Triage algorithms after primary hrHPV screening	SEN, %	SPE, %	PPV, %	NPV, %	Colposcopy referral rate, %	Cytology test rate %	Colposcopies to detect 1 CIN2+
1. None (all HPV positivity to colposcopy)	**93.80**	**87.4**	9.4	99.9	13.7	0.0	10.6
2. ≥ASCUS (comparator)	70.1	97.4	27.3	99.6	3.5	13.7	3.7
3. ≥LSIL	**61.1**	**98.4**	35.1	99.5	2.4	13.7	2.9
4. ≥HSIL	**43.1**	**99.8**	78.5	99.2	0.8	13.7	1.3
5. HPV16+	**46.5**	**98.2**	26.3	99.2	2.4	0.0	3.8
6. HPV16+ and/or 18+	**49.3**	97.5	21.2	99.3	3.2	0.0	4.7
7. HPV16+ and/or ≥ ASCUS	**80.6**	**96.0**	22.1	99.7	5.0	11.3	4.5
8. HPV16+ and ≥ASCUS	**36.1**	**99.5**	52.0	99.1	1.0	11.3	1.9
9. HPV16+ and/or ≥LSIL	74.3	97.2	26.8	99.6	3.8	11.3	3.7
10. HPV16+ and ≥LSIL	**31.3**	**99.8**	64.3	99.1	0.7	11.3	1.6
11. HPV16+ and/or ≥HSIL	64.6	**98.1**	32.3	99.5	2.7	11.3	3.1
12. HPV16+ and ≥HSIL	**22.9**	99.9	84.6	98.9	0.4	11.3	1.2
13. (HPV16+ and/or 18+) and/or ≥ASCUS	**81.3**	**95.4**	19.9	99.7	5.6	10.5	5.0
14. (HPV16+ and/or 18+) and ≥ASCUS	**38.2**	**99.4**	47.4	99.1	1.1	10.5	2.1
15. (HPV16+ and/or 18+) and/or ≥LSIL	75.7	**96.5**	23.1	99.7	4.5	10.5	4.3
16. (HPV16+ and/or 18+) and ≥LSIL	**32.6**	**99.7**	60.3	99.1	0.7	10.5	1.7
17. (HPV16+ and/or 18+) and/or ≥HSIL	66.0	97.4	26.0	99.5	3.5	10.5	3.8
18. (HPV16+ and/or 18+) and ≥HSIL	**24.3**	**99.9**	83.3	99.0	0.4	10.5	1.2

Bold value indicates that the value is significantly different from that of the comparator (p<0.01). SEN, sensitivity; SPE, specificity; ASCUS, atypical squamous cells of undetermined significance; LSIL, low-grade squamous intraepithelial lesion; HSIL, high-grade squamous intraepithelial lesion; CIN2+, cervical intraepithelial neoplasia 2 or worse; HPV, human papillomavirus. PPV, positive predictive value; NPV, negative predictive value.

Notably, of the 17 triage strategies, the one with positivity for HPV16 and/or ≥ASCUS cytology (Strategy 7) and the one with positivity for (HPV16 and/or HPV18) and/or ≥ASCUS cytology (Strategy 13) achieved favorable sensitivity compared with strategy 2 (both 96.3% vs. 96.3% for CIN3+, p = 1.000; 80.6% and 81.3% vs. 70.1% respectively for CIN2+, p <0.001), these algorithms would reduce the colposcopy referral rate to 5.0% and 5.6%, respectively, compared with 13.7% of that for HPV alone.

Furthermore, HPV16 positivity and/or ≥HSIL (Strategy 11) showed an improved specificity of 97.5% (95%CI = 97.2–97.8) at a similar sensitivity of 92.6% (95%CI = 82.1–97.9) for CIN3+ relative to the comparator, and achieved a referral rate of 2.9% and 6.1 colposcopies to detect one CIN3+. These findings were replicated for the endpoint of CIN2+. In addition, HPV16 positivity and/or ≥LSIL cytology (Strategy 9) showed a similar specificity and a comparable sensitivity for detecting CIN2+/CIN3+ to the comparator. However, (HPV16 and/or 18 positivity) and/or ≥LSIL as triage (Strategy 15) revealed comparable sensitivities and worse specificities for detecting CIN2+/CIN3+ compared with the comparator.

Strategy 3 raised the threshold to LSIL, yielding a favorable specificity and a comparable sensitivity for detection of CIN3+ to the comparator, but leading to a worse sensitivity for detection of CIN2+ with misdiagnosis of more high-grade diseases. None of the other strategies showed improvement in specificity without a decreased sensitivity for the detection of CIN2+/CIN3+ as compared to the comparator. Among them, HPV16 alone or HPV16/18 positivity, yielded lower sensitivities for the detection of CIN3+ than the comparator (66.7% and 68.5% vs. 96.3% respectively, p <0.0001).

## Discussion

In the current study, the prevalence rate of hrHPV was 13.7% on self-collected samples. The high prevalence of hrHPV not only leads to unnecessary colposcopy or follow-up burden[9], but also might add to the mental burden of the HPV-positive women[17]. However, in HPV-based screening programs, there is still no consensus on the optimal management of HPV-positive women to maximize disease detection and minimize colposcopy rates [18, 19].

Risk stratification has important implications for the personalized risk management of HPV positive women. For instance, optimizing triage strategies to decide whether referring the patients to immediate colposcopy or follow up[9]. Even after a woman is referred to colposcopy, knowing risk estimates might guide the biopsies taken[20]. In this study, HPV16 infection conferred the highest absolute risks among all genotypes for CIN2+/CIN3+ in overall population and all age groups, which is in consistent with the results of ATHENA trial[21]. However, although the lower overall absolute risk of HPV18 than Other hrHPV in this study, HPV18 is of particular significance due to its association with difficult-to-detect glandular lesions and cancers[22]. Therefore, HPV16 alone or combined with HPV18 could be considered as a maker to personalize triage plans after primary HPV screening[11].

Morphological cytology and molecular HPV genotypes are two most common triages used in clinical practice[23]. Different triage methods compared in our study were similar with those from the ATHENA trial conducted in America[21] and PROHTECT-3B study in the Netherlands by offering self-sampling[21, 24]. The overall and type-specific HPV prevalence among different populations vary greatly by geographic region, ethnic diversity and level of income[25]. For example, a relatively high contribution of HPV58 to cervical cancer in East Asia has been reported [16]. In light of these facts, it is questionable whether similar strategies would be effective in the Chinese population. To our knowledge, there was little study to evaluate the performance of such triage strategies on HPV-positive self-samples in China, particularly large population-based studies. Accordingly, among 10,498 screened women, we assessed the performance of 17 sequential triage algorithms based on HPV16/18 or HPV16 combined with different thresholds of cytology, for the detection of cervical diseases in order to extend the available options for management of hrHPV positive women on self-collected samples since no single strategy that is suitable across all circumstances.

Although several strategies such as dual staining and methylation makers are under consideration in some countries, “cytology ≥ASCUS” is currently one of the most common triage strategies in clinical practice[9, 13], and was performed as the comparator. Similar with prior studies[21, 24], on the basis of our findings, HPV16 (not HPV16/18) combined with a raised threshold of abnormal cytology (≥HSIL) achieve a good performance with favorable specificity and comparable sensitivity versus the comparator. As a result, the colposcopy referral rate decreases from 3.5% to 2.7%; and instead of 7.1 colposcopies, only 5.9 colposcopies needed for detection of one CIN3+. Consequently, with less requirement for colposcopy referral and cytology testing compared with the comparator, this strategy may be more suitable for young and fertile women for whom overtreatment may be harmful in future fertility and pregnancies, while less aggressive detection and management of high-grade lesions may be needed [26]. In addition, there might be a need to reduce the colposcopy rate because of risk of overdiagnosis, high costs, and unnecessary anxiety for the women involved. Furthermore, a watchful waiting policy could be considered for a case of CIN2 since a significant proportion of lesions will regress spontaneously, particularly in young and fertile women[24].

The medical resources required and health care professionals, as well as healthy perceptions of women, are key factors that should be addressed to ensure effective cervical screening programs [27]. Of the 17 triage strategies, the one with positivity for HPV16 and/or ≥ASCUS and the one with positivity for (HPV16 and/or HPV18) and/or ≥ASCUS achieved favorable sensitivity and might reduce secondly loss to follow-up rate compared with the comparator; although with a little lower specificity compared with the comparator, these algorithms would reduce the referral rate to 5.0% and 5.6% respectively compared with 13.7% of that for HPV alone, and require less cytological test compare with the comparator. Since women in low-resource areas are less likely to contact with health care services, early detection of cervical cancer and precancer will generally produce a better outcome for women’s overall health as well as public health in the long run [27]. Moreover, screening algorithms with low sensitivity require frequent repetitions of cervical screening, which pose logistical barriers to adherence to follow-up advices for women in rural areas as well as inequity in access to services [27]. Accordingly, when there is a clinical need to use a test with high potential sensitivity, such as women in low-resource settings where access to appropriate follow-up is challenging and women with serious stress or anxiety on HPV infection, these two strategies (HPV16 and/or ≥ASCUS; HPV16/18 and/or ≥ASCUS) may be appropriate alternatives to achieve the goal of preventing cervical cancer. They targeted a balance between achieving favorable sensitivity relative to the comparator and reducing substantial colposcopy referral compared to HPV testing alone.

Of the 17 triage strategies, combining HPV16 positivity and/or LSIL or worse achieved similar specificity and comparable sensitivity for detection of CIN2+/CIN3+ to the comparator. However, a prior study [24] showed that HPV16 and/or LSIL cytology as triage achieve favorable specificity and comparable sensitivity. Additionally, although the high risk of HPV16/18 contributed to cervical cancer, HPV16/18 couldn’t be used as a sole triage since its low sensitivity, which is consistent with prior studies[21, 24].

One limitation of this substudy is that there might be a verification bias since not all the women underwent biopsy, for instance, some women negative of HPV didn’t meet the requirements of colposcopy referring. However, several HPV assays including cobas4800 and Seq HPV either on self-collected or clinician-collected samples were performed, and all the hrHPV-positive women were suggested to be referred to colposcopy-guided biopsy according to standard POI protocol as well as a high compliance rate of colposcopy referral in HPV-positive women [5], which reduces misdiagnosis, hence the majority of disease was detected in the study population. Moreover, all the slides were reviewed by the senior pathologists in PUSH and subjected to quality control, which makes the results more accuracy and comparable. A major strength of the current study is that it is derived from CHUMUST with a large sample size of natural population from six settings in China, which reflects the diverse demography of Chinese population. Therefore, the findings above may be applicable to routine practice and generalizable to populations with similar characteristics.

Efficient cancer prevention is based on detecting and treating precancers before they become cancer. Implementing this effectively and still avoiding overtreatment should be the primary goal of cervical cancer screening[9]. In several studies, the approach of HPV primary screening and reflex with genotyping and cytology, despite the high colposcopy referral rate, was still found to be the most cost-effective relative to other strategies, including cytology-based screening, co-testing of HPV and cytology, and HPV primary screening with reflex to cytology[10, 21, 24, 27]. There is no single screening modality that could cover all clinical needs; on the basis of our findings and prior studies[24, 28], combination HPV16 or HPV18, with different thresholds of cytology offers personalized management for HPV-positive women in clinical practice.

In conclusion, the study revealed that combinations of HPV16 and the threshold HSIL improves the colposcopy referral rate, cytology testing rate and the specificity for detecting CIN3+ lesions while maintaining adequate sensitivity compared with the triage of cytology ≥ASCUS, thus could be used as a preferred screening strategy for young and fertile women. Combinations of HPV16 or HPV16/18 with ASCUS or worse could improve sensitivity and cytology rate compared with triage of cytology ≥ASCUS, and reduce substantial colposcopy referrals compared with HPV testing alone. Sequential triage algorithms that combinations of HPV16 or HPV16/18 and different thresholds of cytology provide guidance for individualized management of HPV-positive women in clinical practice, and offer practical alternatives for organizing HPV-based screening programs, particularly in low-resource areas.

## Supporting information

S1 File (XLSX)

S1 Fig (TIF)
