# Supplementary figures and images for: The effectiveness of HPV16 and HPV18 genotyping and cytology with different thresholds for the triage of human papillomavirus-based screening on self-collected samples
Source: PLoS One. 2020 Jun 11;15(6):e0234518. doi: 10.1371/journal.pone.0234518 (PMC7289398; doi:10.1371/journal.pone.0234518)

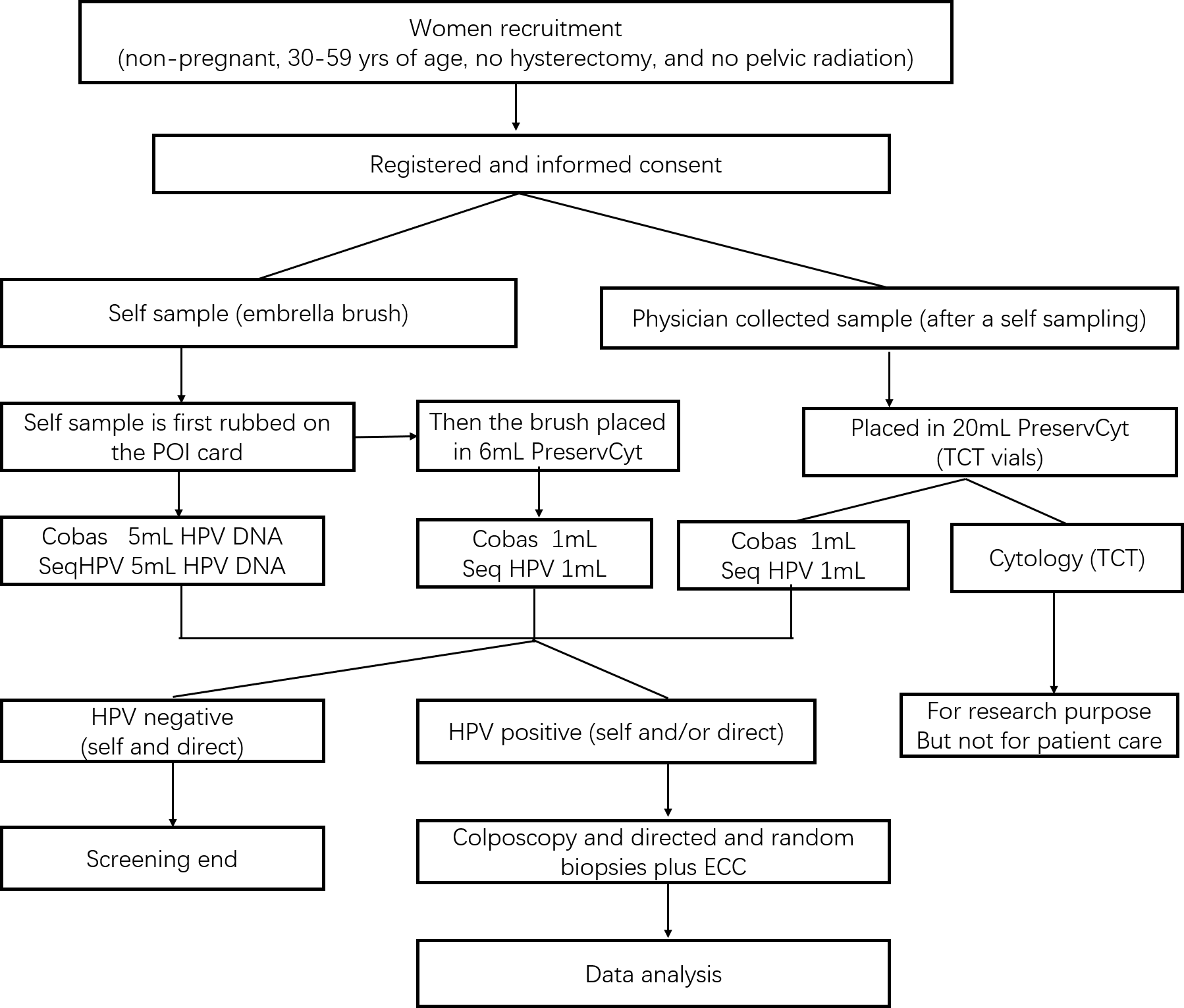

Supplement: S1 Fig — (TIF) [file pone.0234518.s002.tif]
